# Regulatory effects of dexamethasone on NK and T cell immunity

**DOI:** 10.1007/s10787-017-0418-0

**Published:** 2017-11-20

**Authors:** Liying Chen, Mikael Jondal, Konstantin Yakimchuk

**Affiliations:** 1 Department of Microbiology, Tumor and Cell Biology, Karolinska Institutet, 171 77 Stockhom, Sweden; 2 Department of Biosciences and Nutrition, Karolinska Institutet, Novum, 141 83 Huddinge, Sweden

**Keywords:** Glucocorticoids, Dexamethasone, NK cells, T cells, Regulatory T cells

## Abstract

Glucocorticoids (GCs) act via the intracellular glucocorticoid receptor (GR), which can regulate the expression of target genes. With regard to the immune system, GCs may affect both innate and adaptive immunity. Our study analyzed the immunoregulatory effects of dexamethasone (Dex) treatment on splenic T, Treg, NK and NKT cells by treating C57Bl6 mice with various doses of Dex. We observed that treatment with Dex decreased the number of NK cells in the spleen and suppressed their activity. In particular, the expression of both Ly49G and NKG2D receptors was decreased by Dex. However, Dex did not affect the population of NKT cells. With regard to splenic T cells, our results show a dose-dependent reduction in CD3^+^, CD4^+^, CD8^+^, CD44^+^ and CD8^+^CD122^+^ T cells, but a stimulatory effect on CD4^+^CD25^+^ regulatory T cells by Dex treatment. In addition, treatment with Dex suppressed anti-tumor immune response in a mouse EG7 tumor model. We conclude that Dex may suppress both T- and NK-mediated immunity.

## Introduction

Glucocorticoids (GC) affect the immune system by both inhibiting and activating pro-inflammatory and anti-inflammatory cytokines and chemokines. Multiple studies have shown that GCs have potent anti-inflammatory and immunosuppressive properties (Ayroldi et al. 2012; Borghetti et al. 2009; Coutinho and Chapman 2011; Dhabhar 2008, 2009) and are therefore widely used in clinical medicine (Strehl and Buttgereit 2013). To mediate these effects, GCs form a complex with the intracellular glucocorticoid receptor (GR), which can regulate the expression of number of target genes and also acts through other molecular mechanisms (Lu and Cidlowski 2006; Meijsing 2015; Petta et al. 2016; Vandevyver et al. 2014). In addition to genomic mechanisms, GCs also elicit rapid effects mediated via the cell membrane, including regulation of signaling pathways (Croxtall et al. 2000). Many studies have shown that GCs may regulate the functions of various immune cell types affecting both innate and adaptive immunity (Oppong and Cato 2015).

With regard to the innate immune system, GCs were shown to suppress bovine neutrophil phagocytic function (Alabdullah et al. 2015; Diez-Fraile et al. 2000) and inhibit activation of mouse macrophages (Chinenov et al. 2012; Tuckermann et al. 2007). However, the effects of GCs on NK cells of both human and animal origin were not extensively studied. Previous studies of the effects of GCs on NK cells showed controversial results. In particular, GCs were shown to suppress activities of NK cells (Kiecolt-Glaser et al. 1987). In contrast, other studies did not observe any significant effect on survival of NK cells by dexamethasone (Dex) treatment (Kumai et al. 2016). Moreover, GCs were recently shown to epigenetically suppress NK cell lytic activity (Eddy et al. 2014).

In contrast to NK cells, the effects of GCs in T cells included induction of apoptosis and suppression of cytokine production (Ashwell et al. 2000; Herold et al. 2006). With regard to T cell subsets, GCs were shown to participate in the differentiation of T helper (Th) cells (Daynes and Araneo 1989). Moreover, GCs may also affect the pattern of cytokines regulating the differentiation of T cell subsets (Elenkov 2004; Flammer and Rogatsky 2011). Furthermore, several studies demonstrated that GCs suppress the secretion of Th type 2 (Th2) cytokines by human T cells (Rolfe et al. 1992; Wu et al. 1991). Furthermore, recent studies demonstrate that the subsets of T cells show different GC sensitivity (Banuelos and Lu 2016). In addition, our previous study demonstrated that lck-GR mice, overexpressing a transgenic GR in both T cells, have decreased CD4^+^ and CD8^+^ T cell subpopulations (Yakimchuk et al. 2015).

To investigate the effects of GCs on the immune system, we selected Dex, a synthetic GC with high immunosuppressive activity (Mager et al. 2003; Rhen and Cidlowski 2005). Our study analyzed the effects of Dex treatment on both NK and T cell immunity. Our study showed that Dex suppresses both NK and T cells in a dose-dependent manner. In addition, we demonstrated stimulatory effect of Dex on CD4^+^CD25^+^ regulatory T cells (Tregs) and suppressive effect on CD8^+^CD122^+^ Tregs.

## Materials and methods

### Mice and cell lines

Wild-type male C57Bl6 (B6) and the TCR-transgenic OT-1 Rag^−/−^ (OT-1 for short) mice were bred and kept in the animal facility at the Department of Microbiology, Tumor and Cell Biology of Karolinska Institutet, Solna. All mice were 8–10 weeks old and age-matched. Animal experiments were evaluated and approved by the local Ethical Committee for Research on Animals (ethical permit number 382/09).

The study used EG7 cells derived from chicken ovalbumin (OVA)-transfected EL4 cells (a DMBA-induced thymoma cell line) (Zhou et al. 1992). EG7 cells express an OVA peptide (SIINFEKL) epitope on H-2 Kb, recognized by the TCR-transgenic OT-1 mice. EG7 were grown in RPMI 1640 medium, supplemented with 10% heat-inactivated fetal bovine serum, 2 nM l-glutamine, 100 U/ml of penicillin and 100 µg/ml streptomycin.

### Reagents

Dexamethasone (Dex) was obtained from Sigma-Aldrich (Sigma-Aldrich, St. Louis, MO, USA). Fluorochrome-labeled antibodies against CD3, CD4, CD8, CD25, CD44, NK1.1, CD11b, CD27, Ly49D, Ly49G2, Ly49C, NKG2D and NKp46 were purchased from eBioscience (eBioscience Inc., San Diego, CA, USA).

### Treatments and flow cytometry

The mice were treated with 0.1, 1, 10 or 100 µg of Dex in a final concentration of 5% dimethyl sulfoxide (DMSO) in phosphate-buffered saline (PBS)/animal or vehicle by intraperitoneal injections for 3 consecutive days. Spleens were taken 48 h after treatment with Dex. For flow cytometry, the isolated splenocytes were washed with cold PBS in FACS falcon tubes and PBS-diluted antibodies were added directly in the cell pellets. Cells were kept on ice for at least 30 min followed by washing. After staining, cells will be maintained in 1% formaldehyde before the analysis of the samples by flow cytometry. For the intracellular staining, cell pellets were first incubated fixation/permeabilization buffer (BD Pharmingen, Franklin Lakes, NJ, USA) on ice for 30 min. Antibodies were diluted using the permeabilization buffer. Data were processed by the software CellQuest Pro and Summit in the Department of Microbiology, Tumor and Cell Biology Core Facility of Karolinska Institutet.

### The EG7 tumor model

The OT-1 Rag^−/−^ mice were treated with Dex (10 µg/mouse/day) ip for 3 days prior to the engraftment of tumor EG7 cells (Fig. 6a). After 1 week, growing-phase EG7 tumor cells were resuspended in cold PBS and engrafted subcutaneously in the right flank of the age-matched male OT-1 Rag^−/−^ mice. Tumors were measured three times per week using a caliper. The tumor volume was calculated according to formula: width^2^ × length × 0.5. The treatment experiments were terminated when EG7 tumors reached the upper size limit allowed by the ethical permit (1.5 cm^3^).

## Results

### Treatment with Dex modulates NK cells

**Fig. 1:**
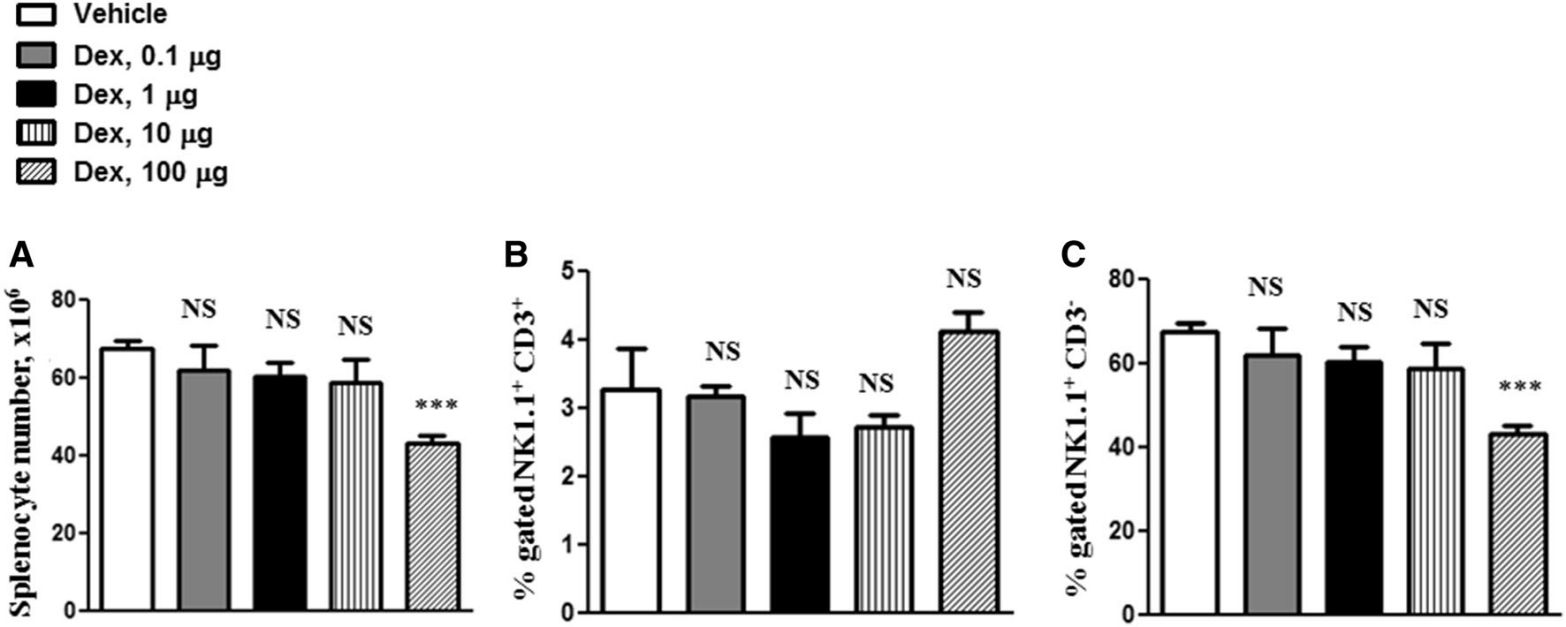
Effects of Dex treatment on NKT and NK cells Splenocytes were isolated from the mice treated with 100, 10, 1 and 0.1 µg of Dex or vehicle (**a**). Splenic NK1.1^+^CD3^+^ NKT and NK1.1^+^CD3^−^ NK cells were isolated at 48 h after treatment with Dex and analyzed by flow cytometry. The results are presented in percentages of NK1.1^+^CD3^+^ NKT (**b**) and NK1.1^+^CD3^−^ NK (**c**) cells. Error bars indicate ± SEM, ***P < 0.001. *NS* not significant. Data are representative of two independent experiments

For analysis, splenocytes were isolated from the mice treated with 0.1, 1, 10 or 100 µg of Dex or vehicle. Absolute splenocyte numbers were significantly reduced by Dex treatment (Fig. 1a). We observed no effects of Dex on NKT cells (Fig. 1b). A moderate suppression of NK cells was observed in mice treated with 100 µg Dex/mouse (Fig. 1c).

**Fig. 2:**
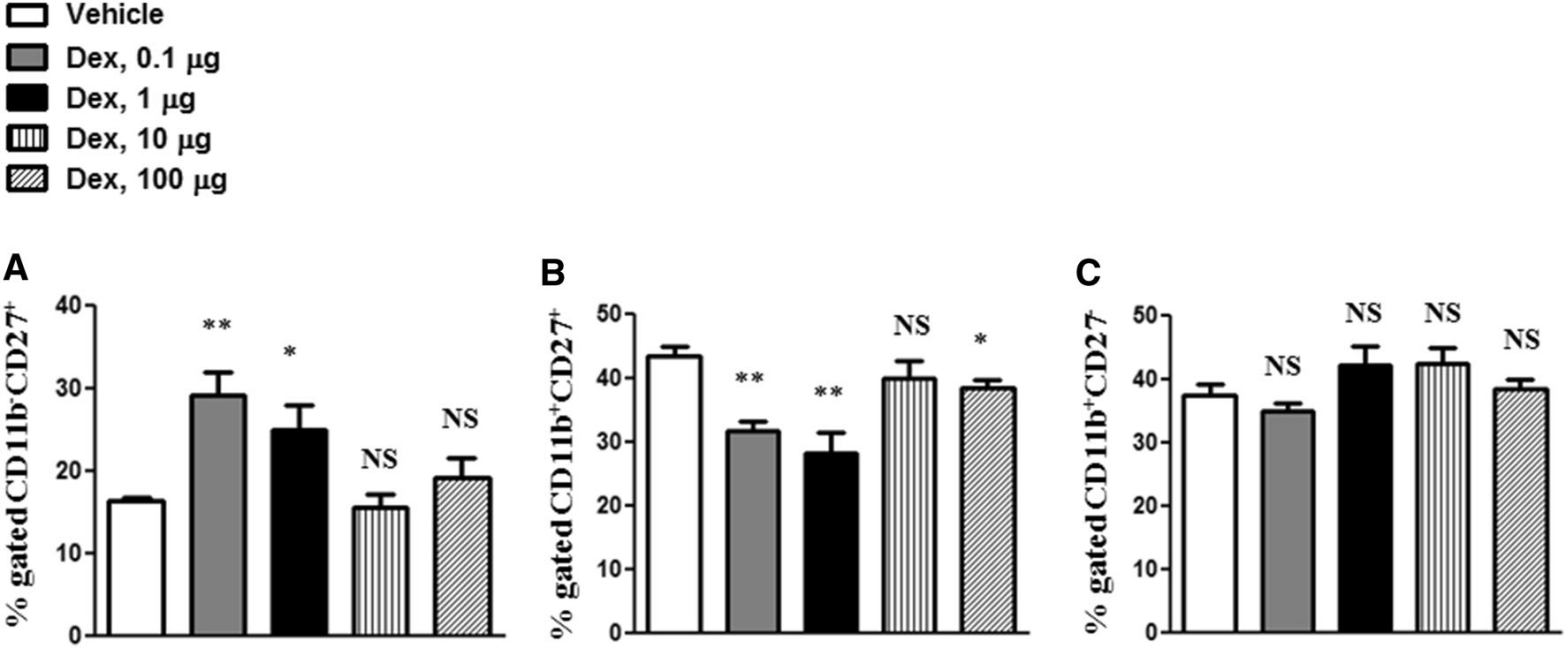
Effects of Dex treatment on NK cell subpopulations NK cell subpopulations in spleen: CD11b^−^CD27^+^, CD11b^+^CD27^+^, CD11b^+^CD27^−^ were analyzed by flow cytometry. The results are presented in percentages of CD11b^−^CD27^+^ (**a**), CD11b^+^CD27^+^ (**b**), CD11b^+^CD27^−^ (**c**) cells. Error bars indicate ± SEM, *P < 0.05, **P < 0.01. *NS* not significant. Data are representative of two independent experiments

To analyze whether Dex may affect the NK cell subpopulations belonging to the different developmental stages of NK cells, the co-expression of CD11b and CD27 markers was evaluated (Fig. 2a–c). Our results showed that the treatment with Dex significantly increased the percentage of CD11b^−^CD27^+^ but decreased the percentage of CD11b^+^CD27^+^ NK cells (Fig. 2a, b).

**Fig. 3:**
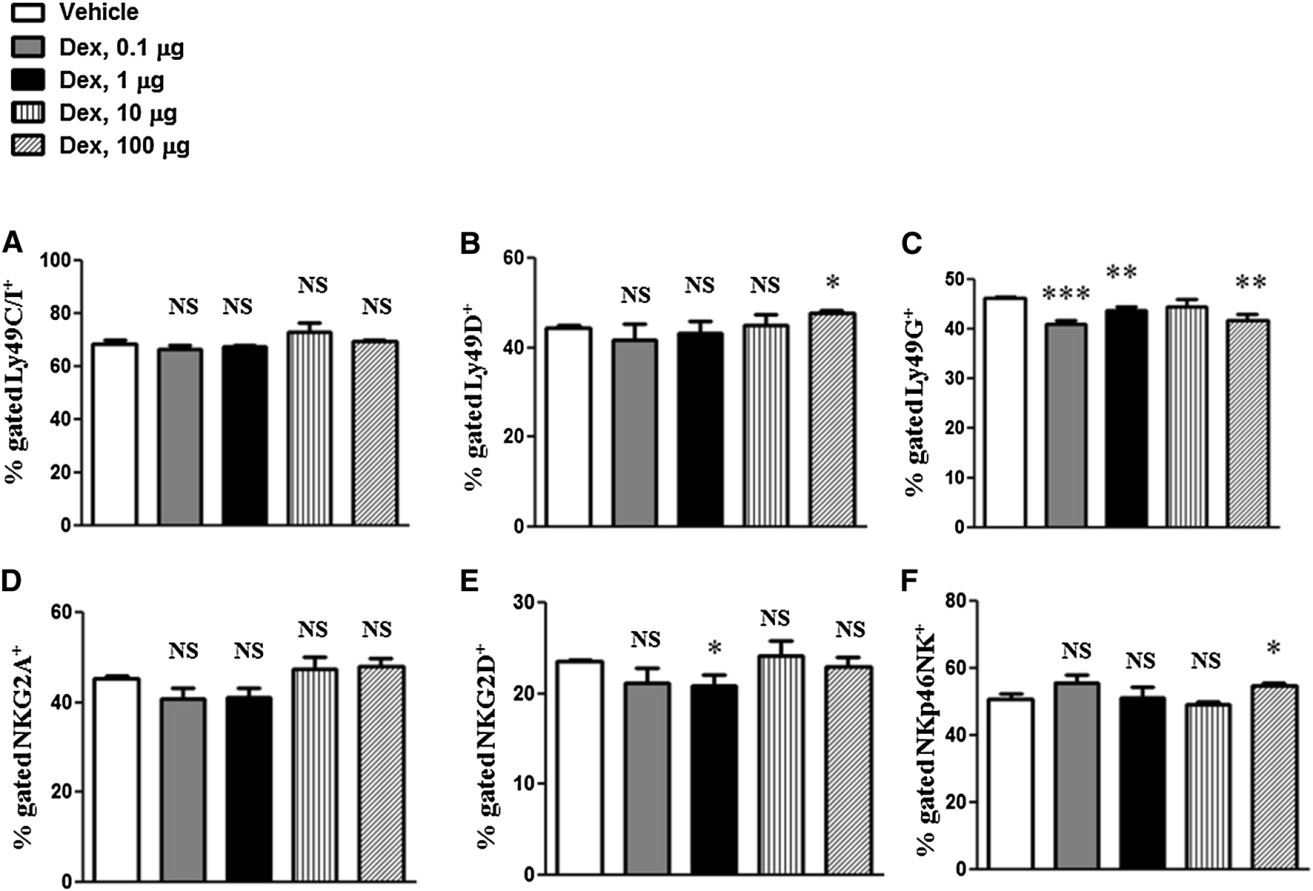
Effects of Dex treatment on the expression of NK cell triggering receptors Expression of NK cell receptors: Ly49C/I^+^ (**a**), Ly49D^+^ (**b**), Ly49G^+^ (**c**), NKG2A^+^ (**d**), NKG2D^+^ (**e**), NKp46NK^+^ (**f**) were analyzed by flow cytometry. The results are presented in percentages of Ly49C/I^+^ (**a**), Ly49D^+^ (**b**), Ly49G^+^ (**c**), NKG2A^+^ (**d**), NKG2D^+^ (**e**), NKp46NK^+^ (**f**) cells. Error bars indicate ± SEM, *P < 0.05, **P < 0.01, ***P < 0.001. *NS* not significant. Data are representative of two independent experiments

To analyze the effects of different doses of Dex on the functional activity of NK cells, we have studied the expression of Ly49 receptors (Fig. 3a–c). We observed the suppressive effects of Dex at doses 1, 10 and 100 µg on the expression of Ly49G (Fig. 3c). In addition, we found moderate suppression of NKG2D and NKp46 at Dex doses of 1 and 100 µg, respectively (Fig. 3e, f).

### Treatment with Dex affects both CD4^+^ and CD8^+^ T cells

**Fig. 4:**
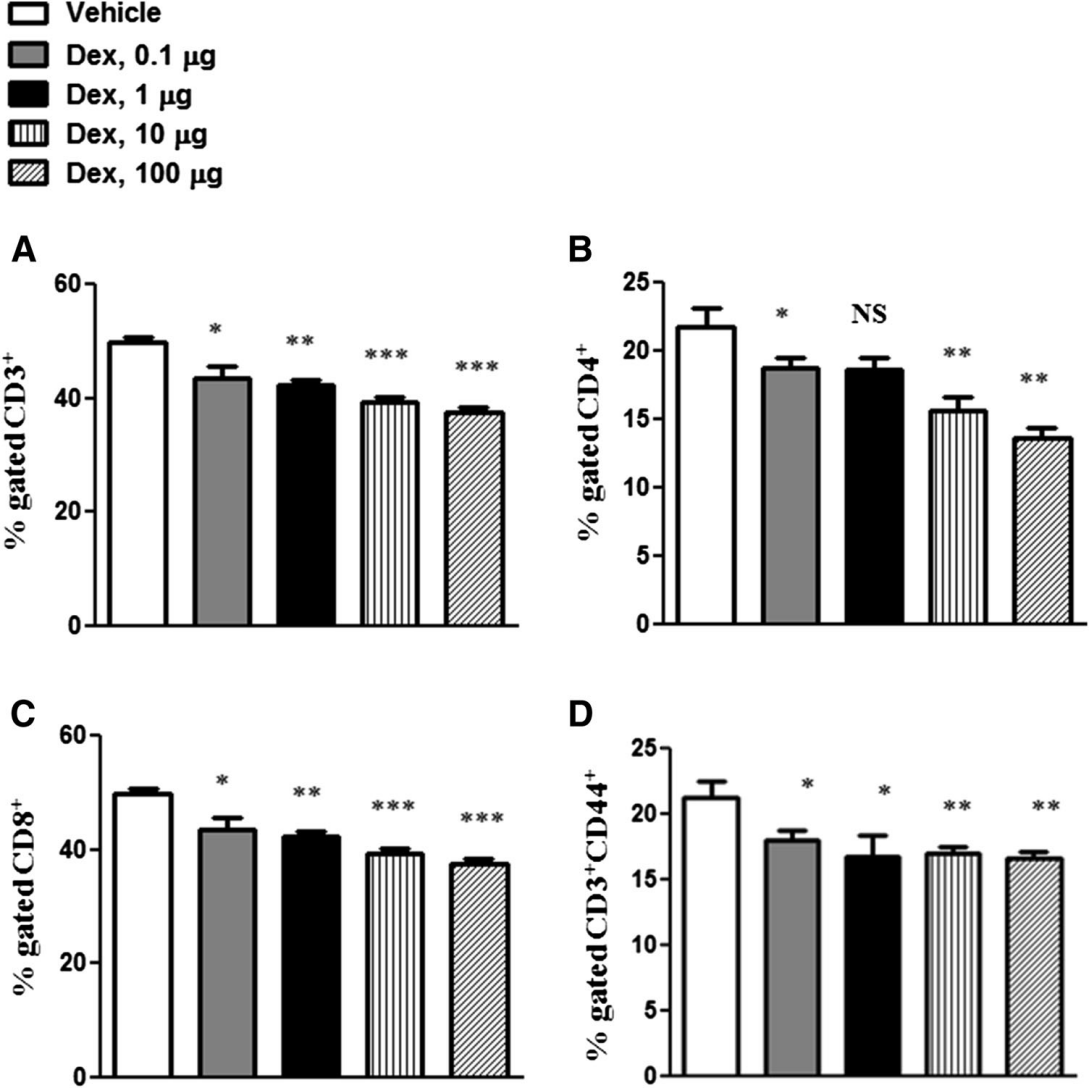
Effects of Dex treatment on CD4^+^ and CD8^+^ T cells T cell subpopulations: CD3^+^ (**a**), CD4^+^ (**b**), CD8^+^ (**c**) and CD44^+^ (**d**) were isolated from spleen at 48 h after treatment with 100, 10, 1 and 0.1 µg of Dex or vehicle and analyzed by flow cytometry. The results are presented in percentages of CD3^+^ (**a**), CD4^+^ (**b**), CD8^+^ (**c**) and CD44^+^ (**d**) cells. Error bars indicate ± SEM, *P < 0.05, **P < 0.01, ***P < 0.001. *NS* not significant. Data are representative of two independent experiments

To test whether GCs affect cell-mediated adaptive immunity, we have analyzed the effects of Dex on different T cell subsets. Treatment with Dex caused dose-dependent reduction in CD3^+^, CD4^+^ and CD8^+^ cells after Dex treatment (Fig. 4a–c). In addition, CD44^+^ T cells, which were shown to belong to central memory T cells, were significantly inhibited by Dex (Fig. 4d).

**Fig. 5:**
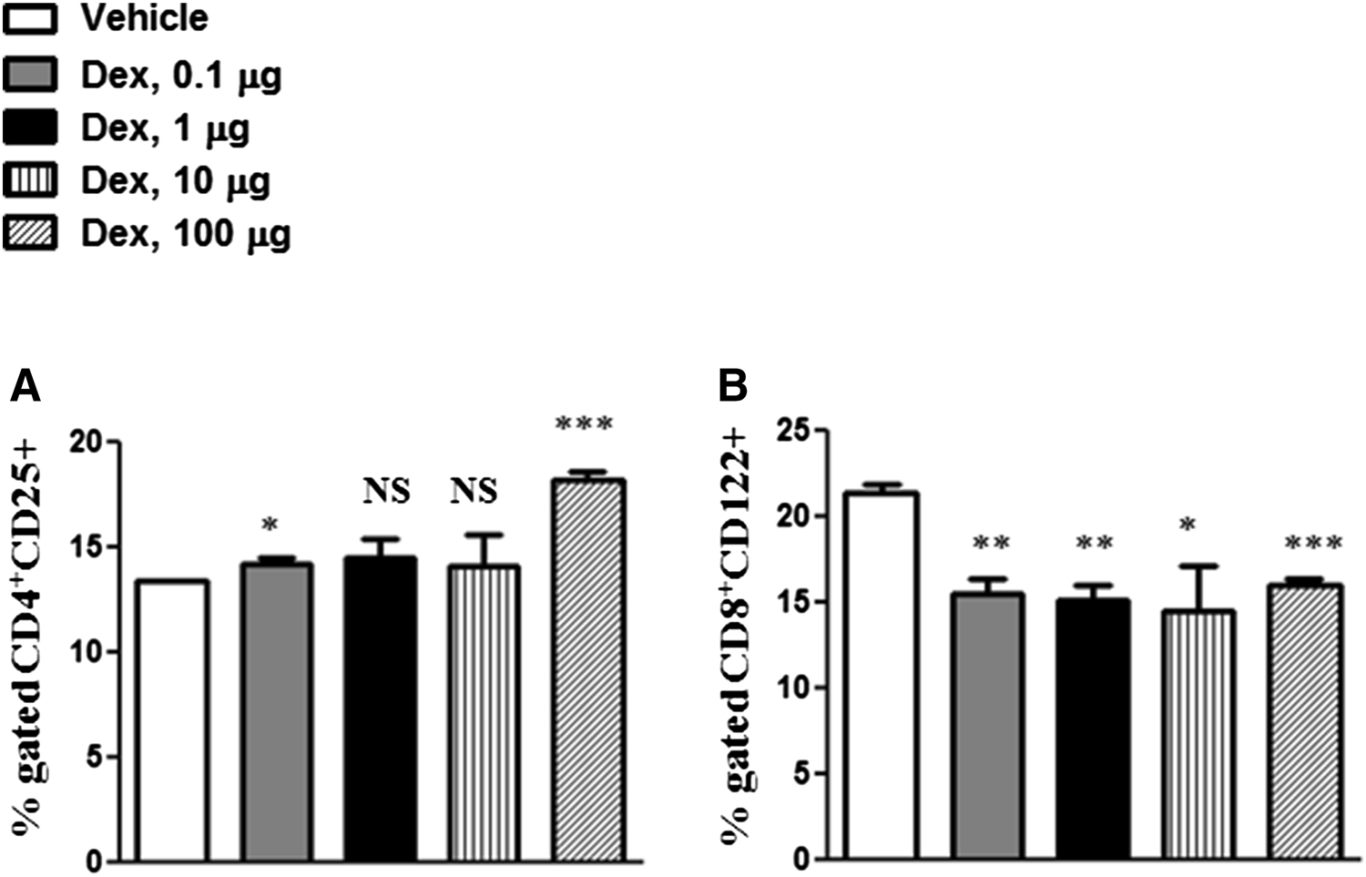
Effects of Dex treatment on regulatory T cells T cell subpopulations: CD4^+^CD25^+^ (**a**) and CD8^+^CD122^+^ (**b**) were analyzed by flow cytometry in splenic T cells. The results are presented in percentages of CD4^+^CD25^+^ (**a**) and CD8^+^CD122^+^ (**b**) cells. Error bars indicate ± SEM, *P < 0.05, **P < 0.01, ***P < 0.001. *NS* not significant. Data are representative of two independent experiments

To evaluate whether Dex may affect subpopulations of Tregs, splenocytes were analyzed by flow cytometry using markers specific for CD4^+^ and CD8^+^ Treg subsets. We observed a significant dose-dependent increase in CD4^+^CD25^+^ Tregs by the treatment with Dex (Fig. 5a). In contrast, treatment with Dex decreased the number of CD8^+^CD122^+^ Tregs (Fig. 5b).

**Fig. 6:**
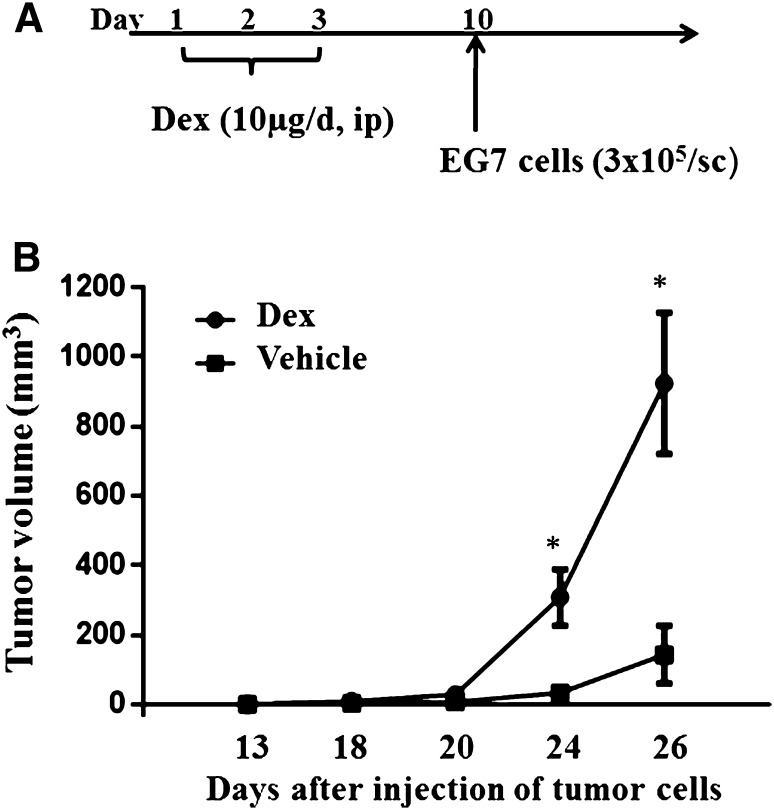
Suppressive effects of Dex on EG7 tumor growth The TCR-transgenic OT-1 Rag^−/−^ were treated with Dex (10 µg/mouse) or vehicle intraperitoneally three times and then subcutaneously injected with EG7 lymphoma cells (**a**). The growth of EG7 tumors in Dex-treated and vehicle-treated mice was analyzed and compared (**b**). Each group consisted of five mice. Error bars indicate ± SEM, *P < 0.05. Data are representative of three independent experiments

To study the effects of GCs on anti-tumor immunity in EG7 tumor model, mice treated with either Dex or vehicle were subcutaneously engrafted with EG7 cells (Fig. 6a). We observed an earlier and faster tumor growth, indicating that EG7 tumors also generated an innate NK response in vivo (Fig. 6b). These results suggest that EG7 tumor induces both an early NK-mediated anti-tumor effect and a late Ag-specific T cell response in vivo.

## Discussion and conclusions

Our study evaluated possible effects of Dex treatment on splenic NKT, NK and T cell subsets. The doses of Dex in our study correspond to the doses used in clinical practice (Czock et al. 2005). With regard to NKT cells, we did not observe any significant effects of different doses of Dex on NK1.1^+^ CD3^+^ cells. This result is in line with previous observations, stating that NKT cells are resistant to Dex (Milner et al. 1999). We detected the moderate inhibitory effects of Dex on NK cells, demonstrated by the decrease in NK1.1^+^CD3^−^ cells. Previously, GCs were demonstrated to prevent IL-15-mediated suppression of NK cells (Moustaki et al. 2011). Also, GCs were shown to have moderate stimulatory effects on the expression of NKG2D and NKp30 in most treated patients (Kumai et al. 2016). In addition, our results indicate that Dex increases the percentage of CD11b^−^CD27^+^ but decreases the percentage of CD11b^+^CD27^+^ NK cells. CD11b^low^CD27^low^ are considered to be the immature stage, while CD11b^high^CD27^high^ belong to the mature stage of NK cell development (Chiossone et al. 2009). These results show that Dex treatment may shift NK cell subpopulations toward an immature stage.

To evaluate the effects of GCs on the activity of NK cells, we have analyzed the expression of both Ly49 inhibitory and triggering receptors on NK cells from the mice treated with different doses of Dex. In particular, Ly49G^+^ NK cells were demonstrated to regulate early NK cell-mediated responses (Barao et al. 2011). We found moderate suppressive effects of Dex by both low and high doses on Ly49G^+^ NK cell subpopulation. In addition to Ly49 receptors, we have studied the expression of the main NK receptors which were previously shown to trigger NK cytotoxicity mediated via interaction between NK and target cells (Bauer et al. 1999). With regard to the triggering receptors, we observed no effect on the expression of NKG2A, but a moderate decrease in the expression of both NKG2D and NKp46 by treatment with 10 μg of Dex. The observed inhibitory effect of Dex on NKp46 is in line with a previous report, demonstrating reduction in NKp46 by methylprednisolone (Vitale et al. 2004).

In addition to the effects on NK cells, our study demonstrates a dose-dependent reduction in both CD4^+^ and CD8^+^ T cells by Dex treatment. Furthermore, the expression of CD44 protein was also significantly reduced in a dose-dependent manner. Previous studies suggested CD44 to be a marker of memory T cells and to be involved in activation and differentiation of T cells (Baaten et al. 2010a, b).

With regard to Tregs, we observed stimulatory effects of GCs on CD4^+^CD25^+^ T cells. This finding is in line with previous studies, demonstrating that GC treatment stimulates regulatory functions of CD4^+^ T cells (Brinkmann and Kristofic 1995). Also, earlier studies have shown that GCs induce the expression of FoxP3 and IL-10 by CD4^+^CD25^+^ T cells (Karagiannidis et al. 2004). Moreover, Dex was demonstrated to elevate the number of CD4^+^CD25^+^ Tregs in peripheral blood and lymphoid organs (Chen et al. 2004, 2006). In addition, several clinical studies reported a positive correlation between treatment with GCs and increase in Tregs in patients with autoimmune diseases (Azab et al. 2008; Braitch et al. 2009; Ling et al. 2007; Suarez et al. 2006).

In addition to the effects on CD4^+^CD25^+^ T cells, we have analyzed the potential influence of Dex on CD8^+^CD122^+^ Tregs. Studies using animal models demonstrated that CD8^+^CD122^+^ Tregs could suppress other autoimmune diseases in animal models. Our results showed that CD8^+^CD122^+^ T cells were suppressed by Dex treatment. Moreover, CD44^+^ T cells, which were shown to belong to memory T cells (Rosenblum et al. 2016), were significantly inhibited by Dex.

Moreover, treatment with Dex significantly inhibited anti-tumor immune response. In our study, faster tumor growth was observed in the mice treated with Dex in comparison with the control mice. This observation may be explained by our present findings showing the suppressive effects of Dex on both T and NK cells. Moreover, it is in line with our previously published study showing faster tumor growth in transgenic mice with overexpression of GR in both T and NK cells (Yakimchuk et al. 2015). In addition, stimulatory effects of GCs on Tregs may affect tumor growth, since Tregs were demonstrated to inhibit anti-tumor response and depletion of Tregs potentiates anti-tumor immune reactions (Dannull et al. 2005; Litzinger et al. 2007).

In conclusion, our study shows that treatment with Dex negatively affects the numbers of both NK and T cells. These results suggest that GCs may suppress both innate and adaptive immunity. Also, we demonstrate that Dex elicits opposite effects on different subpopulations of Tregs by stimulating CD4^+^CD25^+^ T cells but inhibiting CD8^+^CD122^+^ T cells. These findings will help further understanding of the complexity of GC actions on the immune responses and optimizing new therapies involving GCs.

## References

[CR1] Alabdullah HA, Fox LK, Gay JM, Barrington GM, Mealey RH (2015). Effects of dexamethasone and *Mycoplasma bovis* on bovine neutrophil function in vitro. Vet Immunol Immunopathol.

[CR2] Ashwell JD, Lu FW, Vacchio MS (2000). Glucocorticoids in T cell development and function*. Annu Rev Immunol.

[CR3] Ayroldi E, Cannarile L, Migliorati G, Nocentini G, Delfino DV, Riccardi C (2012). Mechanisms of the anti-inflammatory effects of glucocorticoids: genomic and nongenomic interference with MAPK signaling pathways. FASEB J.

[CR4] Azab NA, Bassyouni IH, Emad Y, Abd El-Wahab GA, Hamdy G, Mashahit MA (2008). CD4^+^CD25^+^ regulatory T cells (TREG) in systemic lupus erythematosus (SLE) patients: the possible influence of treatment with corticosteroids. Clin Immunol.

[CR5] Baaten BJ, Li CR, Bradley LM (2010). Multifaceted regulation of T cells by CD44. Commun Integr Biol.

[CR6] Baaten BJ, Li CR, Deiro MF, Lin MM, Linton PJ, Bradley LM (2010). CD44 regulates survival and memory development in Th1 cells. Immunity.

[CR7] Banuelos J, Lu NZ (2016). A gradient of glucocorticoid sensitivity among helper T cell cytokines. Cytokine Growth Factor Rev.

[CR8] Barao I (2011). Mouse Ly49G2^+^ NK cells dominate early responses during both immune reconstitution and activation independently of MHC. Blood.

[CR9] Bauer S, Groh V, Wu J, Steinle A, Phillips JH, Lanier LL, Spies T (1999). Activation of NK cells and T cells by NKG2D, a receptor for stress-inducible MICA. Science.

[CR10] Borghetti P, Saleri R, Mocchegiani E, Corradi A, Martelli P (2009). Infection, immunity and the neuroendocrine response. Vet Immunol Immunopathol.

[CR11] Braitch M, Harikrishnan S, Robins RA, Nichols C, Fahey AJ, Showe L, Constantinescu CS (2009). Glucocorticoids increase CD4CD25 cell percentage and Foxp3 expression in patients with multiple sclerosis. Acta Neurol Scand.

[CR12] Brinkmann V, Kristofic C (1995). Regulation by corticosteroids of Th1 and Th2 cytokine production in human CD4^+^ effector T cells generated from CD45RO- and CD45RO^+^ subsets. J Immunol.

[CR13] Chen X, Murakami T, Oppenheim JJ, Howard OM (2004). Differential response of murine CD4^+^CD25^+^ and CD4^+^CD25-T cells to dexamethasone-induced cell death. Eur J Immunol.

[CR14] Chen X, Oppenheim JJ, Winkler-Pickett RT, Ortaldo JR, Howard OM (2006). Glucocorticoid amplifies IL-2-dependent expansion of functional FoxP3(+)CD4(+)CD25(+) T regulatory cells in vivo and enhances their capacity to suppress EAE. Eur J Immunol.

[CR15] Chinenov Y, Gupte R, Dobrovolna J, Flammer JR, Liu B, Michelassi FE, Rogatsky I (2012). Role of transcriptional coregulator GRIP1 in the anti-inflammatory actions of glucocorticoids. Proc Natl Acad Sci USA.

[CR16] Chiossone L, Chaix J, Fuseri N, Roth C, Vivier E, Walzer T (2009). Maturation of mouse NK cells is a 4-stage developmental program. Blood.

[CR17] Coutinho AE, Chapman KE (2011). The anti-inflammatory and immunosuppressive effects of glucocorticoids, recent developments and mechanistic insights. Mol Cell Endocrinol.

[CR18] Croxtall JD, Choudhury Q, Flower RJ (2000). Glucocorticoids act within minutes to inhibit recruitment of signalling factors to activated EGF receptors through a receptor-dependent, transcription-independent mechanism. Br J Pharmacol.

[CR19] Czock D, Keller F, Rasche FM, Haussler U (2005). Pharmacokinetics and pharmacodynamics of systemically administered glucocorticoids. Clin Pharmacokinet.

[CR20] Dannull J (2005). Enhancement of vaccine-mediated antitumor immunity in cancer patients after depletion of regulatory T cells. J Clin Investig.

[CR21] Daynes RA, Araneo BA (1989). Contrasting effects of glucocorticoids on the capacity of T cells to produce the growth factors interleukin 2 and interleukin 4. Eur J Immunol.

[CR22] Dhabhar FS (2008). Enhancing versus suppressive effects of stress on immune function: implications for immunoprotection versus immunopathology. Allergy Asthma Clin Immunol.

[CR23] Dhabhar FS (2009). Enhancing versus suppressive effects of stress on immune function: implications for immunoprotection and immunopathology. Neuroimmunomodulation.

[CR24] Diez-Fraile A, Meyer E, Massart-Leen AM, Burvenich C (2000). Effect of isoproterenol and dexamethasone on the lipopolysaccharide induced expression of CD11b on bovine neutrophils. Vet Immunol Immunopathol.

[CR25] Eddy JL, Krukowski K, Janusek L, Mathews HL (2014). Glucocorticoids regulate natural killer cell function epigenetically. Cell Immunol.

[CR26] Elenkov IJ (2004). Glucocorticoids and the Th1/Th2 balance. Ann N Y Acad Sci.

[CR27] Flammer JR, Rogatsky I (2011). Minireview: glucocorticoids in autoimmunity: unexpected targets and mechanisms. Mol Endocrinol.

[CR28] Herold MJ, McPherson KG, Reichardt HM (2006). Glucocorticoids in T cell apoptosis and function. Cell Mol Life Sci CMLS.

[CR29] Karagiannidis C (2004). Glucocorticoids upregulate FOXP3 expression and regulatory T cells in asthma. J Allergy Clin Immunol.

[CR30] Kiecolt-Glaser JK, Glaser R, Shuttleworth EC, Dyer CS, Ogrocki P, Speicher CE (1987). Chronic stress and immunity in family caregivers of Alzheimer’s disease victims. Psychosom Med.

[CR31] Kumai T, Oikawa K, Aoki N, Kimura S, Harabuchi Y, Kobayashi H (2016). Assessment of the change in cetuximab-induced antibody-dependent cellular cytotoxicity activity of natural killer cells by steroid. Head Neck.

[CR33] Ling Y, Cao X, Yu Z, Ruan C (2007). Circulating dendritic cells subsets and CD4^+^ Foxp3^+^ regulatory T cells in adult patients with chronic ITP before and after treatment with high-dose dexamethasome. Eur J Haematol.

[CR34] Litzinger MT, Fernando R, Curiel TJ, Grosenbach DW, Schlom J, Palena C (2007). IL-2 immunotoxin denileukin diftitox reduces regulatory T cells and enhances vaccine-mediated T-cell immunity. Blood.

[CR35] Lu NZ, Cidlowski JA (2006). Glucocorticoid receptor isoforms generate transcription specificity. Trends Cell Biol.

[CR36] Mager DE, Moledina N, Jusko WJ (2003). Relative immunosuppressive potency of therapeutic corticosteroids measured by whole blood lymphocyte proliferation. J Pharm Sci.

[CR37] Meijsing SH (2015). Mechanisms of glucocorticoid-regulated gene transcription. Adv Exp Med Biol.

[CR38] Milner JD, Kent SC, Ashley TA, Wilson SB, Strominger JL, Hafler DA (1999). Differential responses of invariant V alpha 24 J alpha Q T cells and MHC class II-restricted CD4^+^ T cells to dexamethasone. J Immunol.

[CR39] Moustaki A, Argyropoulos KV, Baxevanis CN, Papamichail M, Perez SA (2011). Effect of the simultaneous administration of glucocorticoids and IL-15 on human NK cell phenotype, proliferation and function. Cancer Immunol Immunother CII.

[CR40] Oppong E, Cato AC (2015). Effects of glucocorticoids in the immune system. Adv Exp Med Biol.

[CR41] Petta I, Dejager L, Ballegeer M, Lievens S, Tavernier J, De Bosscher K, Libert C (2016). The interactome of the glucocorticoid receptor and its influence on the actions of glucocorticoids in combatting inflammatory and infectious diseases. Microbiol Mol Biol Rev MMBR.

[CR42] Rhen T, Cidlowski JA (2005). Antiinflammatory action of glucocorticoids—new mechanisms for old drugs. N Engl J Med.

[CR43] Rolfe FG, Hughes JM, Armour CL, Sewell WA (1992). Inhibition of interleukin-5 gene expression by dexamethasone. Immunology.

[CR44] Rosenblum MD, Way SS, Abbas AK (2016). Regulatory T cell memory. Nat Rev Immunol.

[CR45] Strehl C, Buttgereit F (2013). Optimized glucocorticoid therapy: teaching old drugs new tricks. Mol Cell Endocrinol.

[CR46] Suarez A, Lopez P, Gomez J, Gutierrez C (2006). Enrichment of CD4^+^ CD25 high T cell population in patients with systemic lupus erythematosus treated with glucocorticoids. Ann Rheum Dis.

[CR47] Tuckermann JP (2007). Macrophages and neutrophils are the targets for immune suppression by glucocorticoids in contact allergy. J Clin Investig.

[CR48] Vandevyver S, Dejager L, Libert C (2014). Comprehensive overview of the structure and regulation of the glucocorticoid receptor. Endocr Rev.

[CR49] Vitale C (2004). The corticosteroid-induced inhibitory effect on NK cell function reflects down-regulation and/or dysfunction of triggering receptors involved in natural cytotoxicity. Eur J Immunol.

[CR50] Wu CY, Fargeas C, Nakajima T, Delespesse G (1991). Glucocorticoids suppress the production of interleukin 4 by human lymphocytes. Eur J Immunol.

[CR51] Yakimchuk K, Chen L, Hasni MS, Okret S, Jondal M (2015). The selective impact of transgenically expressed glucocorticoid receptor on T cells. Autoimmunity.

[CR52] Zhou F, Rouse BT, Huang L (1992). Prolonged survival of thymoma-bearing mice after vaccination with a soluble protein antigen entrapped in liposomes: a model study. Can Res.

